# Correlates of hepatitis B testing in Ghana: The role of knowledge, stigma endorsement and knowing someone with hepatitis B

**DOI:** 10.1111/hsc.13860

**Published:** 2022-06-14

**Authors:** Charles Ampong Adjei, Sarah E. Stutterheim, Fleuren Bram, Florence Naab, Robert A. C. Ruiter

**Affiliations:** ^1^ Department of Community Health Nursing University of Ghana Accra Ghana; ^2^ Department of Health Promotion Care and Public Health Research Institute, Maastricht University Maastricht The Netherlands; ^3^ Department of Work and Social Psychology Maastricht University Maastricht The Netherlands; ^4^ Department of Maternal and Child Health University of Ghana Accra Ghana

**Keywords:** Ghana, hepatitis B, knowing someone with hepatitis B, knowledge, stigma, testing

## Abstract

Hepatitis B testing is the gateway for prevention and care. However, previous studies document low hepatitis B testing uptake in sub‐Saharan Africa. This study investigated knowledge, stigma endorsement and knowing someone with hepatitis B as correlates of hepatitis B testing behaviours among people in the Greater Accra and Northern regions of Ghana. A cross‐sectional survey was completed by 971 participants (Greater Accra = 503, and Northern region = 468) between October 2018 and January 2019. Approximately 54% of the participants reported having been tested for hepatitis B. The logistic regression analyses showed that having greater hepatitis B knowledge was positively associated with hepatitis B testing (OR = 1.22, 95% CI: 1.14–1.30). Higher hepatitis B stigma endorsement was negatively related to hepatitis B testing (OR = 0.97, 95% CI: 0.96–0.99). Also, participants who knew someone (i.e. parent, sibling and/or friend) with hepatitis B were more likely to have tested compared to those who did not know someone with hepatitis B (OR = 7.15, 95% CI: 5.04–10.14). This study demonstrates that knowing someone with hepatitis B increases the likelihood of testing, highlighting the need to create safe and non‐judgmental contexts for people with hepatitis B (PWHB) to disclose if they want to. Also, given that greater hepatitis B knowledge increases testing and hepatitis B stigma endorsement impedes testing, interventions that increase knowledge and reduce stigma should be incorporated in efforts to promote testing in Ghana.

**What is known about the topic?**

- The burden of hepatitis B is high in Ghana, yet testing uptake remains sub‐optimal.
- Evidence of health‐related factors influencing hepatitis B testing in Ghana has been previously documented but not individual level factors.
- Hepatitis B‐related stigma and knowledge are important determinants of testing.

**What this paper adds?**

- This study adds to the limited number of quantitative studies on the correlates of hepatitis B testing in Africa.
- Individual level factors influencing hepatitis B testing uptake in Ghana is documented using the behavioural model of health service utilisation as a guide.
- The need for stigma reduction intervention is highlighted.

## 1 INTRODUCTION

Hepatitis B viral (HBV) infection remains a significant public health threat worldwide. Globally, about 257 million people are chronically infected with hepatitis B, and 887,000 deaths occur annually due to hepatitis B‐related complications (World Health Organization [WHO], 2019). Africa is disproportionately affected by hepatitis B (Béguelin et al., 2018; Breakwell et al., 2017; McNaughton et al., 2020; Lemoine & Thursz, 2017; Spearman et al., 2017) with a prevalence estimate of 8.83% compared to 5.26% in the Western Pacific region, and 0.20% in the Americas (Schweitzer et al., 2015). The majority of people with hepatitis B (PWHB) in Africa reside in Central and Western Africa (WHO, 2017a). Ghana, a West African country, has a hepatitis B prevalence estimate of 12.3% (Ofori‐Asenso & Agyeman, 2016), far above the 8% threshold for classification of high endemic countries by the World Health Organization (WHO, 2019).

Scaling up hepatitis B testing, and diagnosis is an effective response to the burden of hepatitis B (Dionne‐Odom et al., 2018; Spearman et al., 2017; WHO, 2017b; WHO, 2018). Hepatitis B testing can break the disease transmission cycle (Cochrane et al., 2016; Spearman et al., 2017; WHO, 2017b). It can lead to the early identification of PWHB (Easterbrook et al., 2017), and subsequently link them to care and treatment (Lemoine et al., 2015). Hepatitis B testing also provides an opportunity to link people to interventions that can reduce transmission, including counselling on risk behaviours, the supply of prevention commodities (e.g. sterile needles and syringes) and vaccination (WHO, 2017b). However, in sub‐Saharan Africa (SSA), less than 1% of PWHB are aware of their hepatitis B status (Béguelin et al., 2018). As such, the optimal benefit of hepatitis B testing is yet to be achieved by SSA (Anfaara et al., 2018; Béguelin et al., 2018). Therefore, it is crucial to better understand what makes people get tested for hepatitis B.

In countries other than Ghana, research has identified several psychosocial factors that compromise access to hepatitis B testing (Lemoine et al., 2015; Li et al., 2012; World Hepatitis Alliance, 2018). These include fear of a positive diagnosis, stigma and inadequate knowledge about hepatitis B (Hamdiui et al., 2018; Li et al., 2017; WHO, 2017b; World Hepatitis Alliance, 2018). Regarding hepatitis B knowledge, previous studies have established a gap among different sub‐populations in Ghana (Abdulai et al., 2016; Adjei et al., 2018; Adoba et al., 2015; Kwadzokpui et al., 2020). For example, a study conducted among barbers in the Obuasi municipality of Ghana found that nearly two‐thirds (64.5%) of the participants were unaware of the modes of transmission for hepatitis B (Adoba et al., 2015). Similarly, in a study done in Brong Ahafo region of Ghana, more than half (59%) of the pregnant women included did not know that hepatitis B can be prevented through vaccination (Adjei et al., 2018). What has not yet been investigated in Ghana is the relationship between hepatitis B knowledge and testing. However, evidence from other locales (e.g. Canada and the United States) indicate that higher hepatitis B knowledge is associated with an increase in the uptake of hepatitis B testing (Calderon et al., 2014; Li et al., 2012).

Another factor that has been found to influence hepatitis B testing is stigma (Cochrane et al., 2016; Franklin et al., 2018; Freeland et al., 2020; Hara et al., 2018; Li et al., 2012; Smith‐Palmer et al., 2020). For example, among African migrants in the United States, fear of being stigmatised was negatively related to testing uptake (Sriphanlop et al., 2014). Similarly, more stigma was associated with a decrease in hepatitis B testing among Chinese migrants in a study done in Canada (Li et al., 2012). However, it remains unclear whether these findings also generalise to a Ghanaian context given the cultural differences. Although hepatitis B stigmatisation has been reported in Northern and Southern Ghana (Adjei et al., 2017, 2019), the relationship between hepatitis B stigma and testing has not previously been investigated in the Ghanaian context.

Further, an awareness of PWHB's sero‐status among close family contacts has been identified as an important predictor for hepatitis B testing in some countries including Zambia (Franklin et al., 2018), Canada (Li et al., 2012) and USA (Cheng et al., 2017). However, its role in hepatitis B testing is not known in the Ghanaian context.

To date, the only study that has examined hepatitis B testing in Ghana looked at health facilities as a contextual factor but did not explore individual factors related to hepatitis B testing (Anfaara et al., 2018). In order to better understand the determinants of hepatitis B testing in Ghana, we investigated hepatitis B knowledge, hepatitis B stigma endorsement and knowing someone with hepatitis B as correlates of testing for hepatitis B among Ghanaians.

## 2 THEORETICAL PERSPECTIVE

The theoretical perspective adopted in this study was the behavioural model of health service utilisation (Andersen, 1995). According to the model, utilisation of health services is a function of an individual's predisposition to use a service, enabling or impeding factors and the need for care (Andersen, 1995). Predisposing factors are the characteristics of a person including demographic variables. Enabling and impeding factors are factors capable of either enhancing or inhibiting an individual's use of healthcare services. Lastly, need factors are the immediate triggers to healthcare service utilisation by an individual (Andersen, 1995). Several studies have adopted and applied the behavioural model of health service utilisation to understand different aspects of health service use (Azfredrick, 2016; Li et al.,  2016; Tolera et al., 2020) including the uptake of breast cancer screening (Harcourt et al., 2014; Lee et al., 2020), cervical cancer screening (Brzoska et al., 2020) and hepatitis B screening (Li et al., 2012). Following the conceptualisation of the constructs of the behavioural model of health service utilisation for hepatitis B testing by Li et al. (2012), demographic characteristics of the participants such as age, gender, marital status, educational attainment, religion, place of residence and employment status were considered as predisposing factors. Hepatitis B knowledge and stigma endorsement were considered enabling and impeding factors, respectively (Li et al., 2012). Lastly, knowing someone with hepatitis B was described as a need factor (Li et al., 2012).

## 3 METHODS

### 3.1 Participants

Participants in the study were Ghanaians older than 18 years and reside in the Greater Accra and Northern regions of Ghana. Based on Krejcie and Morgan's (1970) formula for determining sample size, 971 participants (503 from the Greater Accra Region, 468 from the Northern Region) were recruited for the cross‐sectional survey. Greater Accra and the Northern regions were chosen because we wanted a good representation of people from the southern and northern part of the country.

### 3.2 Measures

*Hepatitis B testing* was measured with a single item asking participants whether they have ever tested for hepatitis B. Answers were provided as either yes or no.

*Hepatitis B knowledge* was measured using an existing 15 item index previously used to measure hepatitis B knowledge in Ghana (Adjei et al., 2018). The index elicited questions on hepatitis B modes of transmission, diagnosis, prevention, treatment and follow‐up care. Answers were provided with either yes, no and do not know. Subsequently, the knowledge items were recoded into correct and incorrect responses. A higher score is indicative of a greater knowledge. All items can be seen in Table 2.

*Hepatitis B stigma endorsement* was measured using an adapted version of the 20‐item Toronto Chinese Hepatitis B Stigma Index (Li et al., 2012). To ensure cultural appropriateness, we pretested the stigma index with 25 Ghanaian individuals, and the feedback obtained informed minor adaptation of some of the items. Specifically, we changed the word ‘sweater’ to ‘clothes’ and ‘grocery shop’ to ‘supermarket’. Answers were provided on a 5‐point Likert index ranging from 1 (strongly disagree) to 5 (strongly agree). A higher score is indicative of greater stigma endorsement (Li et al., 2012). The index showed a moderate to good internal consistency in this present study (Cronbach's Alpha value = 0.78). All items can be seen in Table 3.

*Knowing someone with hepatitis B* was measured by the question ‘Do you know someone who is hepatitis B positive?’. Participants were asked to indicate either yes or no and further indicate their relationship with people they knew to have hepatitis B if they answered yes.

*Demographic characteristics* measured included gender, age, marital status, educational attainment, occupation, religion and place of residence.

### 3.3 Procedure

Following approval of this study by the Korle‐Bu Institutional Review Board (KBTH‐IRB), we visited the study areas (i.e. Greater Accra and Northern region) between October 2018 and January 2019. Within these areas, we selected one district each through balloting. To arrive at the precise sample, we selected three electoral areas within each of the districts sampled. With the assistance of the assembly leaders in the electoral areas, we located roundabouts where a pen was spun, and the first house that faced the direction of the tip of the pen was used as the starting point for data collection (Grais et al., 2007). Participants were conveniently selected from the selected areas. The research assistants approached the potential participants in front of their homes mostly in the evening and weekends when many of the residents have returned from work. The purpose of the study was explained to the potential participants (i.e. those who met the inclusion criteria) and those who consented to participate were given a consent form to either sign or thumbprint. Overall, 62% of the participants were able to complete the questionnaire without any support. The research assistants assisted the remaining 38%. None of the participants received any form of compensation for taking part in the study. The response rate was 95%. On the average, participants took 20 minutes to complete the questionnaire.

### 3.4 Data analyses

SPSS version 23.0 was used for the analyses. During data cleaning, retrieved questionnaires were checked for completeness and those found to have greater than 10% missing responses were excluded from the analyses. In the analyses, we first generated descriptive statistics (percentages, frequencies, means, standard deviations to describe the background of the participants). We then examined the responses per item of the questionnaire regarding hepatitis B knowledge and stigma endorsement. The 5‐point Likert stigma index was transformed into 3‐point index. These include disagree (i.e. combination of strongly disagree and disagree), agree (i.e., combination of strongly agree and agree) and neutral (neither agree nor disagree). Third, we used chi‐square test for univariate analyses to examine the association between the categorical demographic variables and hepatitis B testing. Fourth, we included the demographic variables that were found to be significantly related to testing behaviour in the chi‐square tests in the first binary logistic regression model (i.e. marital status and educational attainment). The second binary logistic regression model (Model 2) then consisted of the predictors of interest (i.e. knowledge, stigma endorsement and knowing someone with hepatitis B) complemented with the demographic characteristics considered in Model 1. The level of statistical significance was set at *p* < 0.05. The odds ratio and 95% confidence interval were used to determine factors that were significantly associated with hepatitis B testing.

## 4 RESULTS

### 4.1 Participant's characteristics

In total, 971 participants (i.e. 503 from the Greater Accra Region; 468 from the Northern Region) took part in the cross‐sectional survey. Of these 971, 47.1% were male and 52.9% were female. About three‐quarters (78.8%) had at least a secondary education. Ages ranged from 18 to 51 years (*M* = 30.52, *SD* = 8.10). Table 1 provides sample characteristics in details.

**TABLE 1:** Participant characteristics

Variables	Frequency	Percent
Gender		
Male	457	47.1
Female	514	52.9
Age (years)		
<25	318	32.7
26–35	402	41.4
>35	251	25.9
M ± SD	30.52 (8.10)	
Marital status		
Single	555	57.2
Married	392	40.4
Divorced	13	1.3
Co‐habitation	11	1.1
Level of education		
No formal education	70	7.2
Primary school	134	13.8
Junior high school	252	26
Senior high school	309	31.8
Post‐secondary education	206	21.2
Occupation		
Farming	178	18.3
Trading	275	28.3
Civil servant	261	26.9
Unemployed	200	20.6
Student	57	5.9
Religion		
Christianity	715	73.7
Islam	244	25.1
African Traditional religion	12	1.2
Place of residence		
Greater Accra Region	503	51.8
Northern Region	468	48.2

### 4.2 Hepatitis B testing

Of the 971 participants, 520 (53.6%) had tested for hepatitis B and 451 (46.4%) had not.

### 4.3 Hepatitis B knowledge

Table 2 presents the responses to each of the knowledge items. The items upon which participants most frequently demonstrated incorrect knowledge were: hepatitis B can be transmitted through contact with an infected person's sweat (False; 82.0% incorrect), there are effective treatments for hepatitis B (True; 63.7% incorrect), people infected with hepatitis B may show no symptoms (True; 55.3% incorrect) and hepatitis B can be spread by sharing eating utensils such as a spoon (False; 46.1%). The mean sum score of the knowledge index was 9.69 (*SD* = 2.36) (index range 0–15). In line with examination scores in Ghana (Opoku‐Asare & Siaw, 2015), a total hepatitis B knowledge score of less than 8.0 was considered as poor, 8.0–12.0 considered as fair and greater than 12.0 considered as good. Overall, 29.6% (*n* = 287) had poor knowledge, 41.8% (*n* = 406) had fair knowledge and 26.8% (*n* = 278) had good knowledge.

**TABLE 2:** Hepatitis B knowledge items and scores

		Correct, *n* (%)	Incorrect, *n* (%)
In my opinion, hepatitis B affects the liver	T	797 (82.1)	174 (17.9)
In my opinion, hepatitis B can be transmitted through unprotected sexual contact	T	773 (79.6)	198 (20.4)
In my opinion, hepatitis B can be transmitted from mother to child at birth	T	629 (64.8)	342 (35.2)
In my opinion, hepatitis B can be caused by a curse	F	739 (76.1)	232 (23.9)
In my opinion, hepatitis B can be caused by spiritual poisoning	F	818 (84.2)	153 (15.8)
In my opinion, hepatitis B can be caused by witches/wizards	F	744 (76.6)	227 (23.4)
In my opinion, hepatitis B can cause liver cancer	T	634 (65.3)	337 (34.7)
In my opinion, there are effective treatments for hepatitis B	T	352 (36.3)	619 (63.7)
In my opinion, there is a vaccine to prevent hepatitis B	T	723 (74.5)	248 (25.5)
In my opinion, people infected with hepatitis B may show no symptoms	T	434 (44.7)	537 (55.3)
In my opinion, people with hepatitis B do not need to be followed up by a doctor	F	840 (86.5)	131 (13.5)
In my opinion, hepatitis B can be spread by sharing eating utensils such as a spoon	F	523 (53.9)	448 (46.1)
In my opinion, hepatitis B can be transmitted through contact with an infected person's sweat	F	175 (18.0)	796 (82.0)
In my opinion, hepatitis B carriers can only be identified by a blood test	T	643 (66.2)	328 (33.8)
Babies born from mothers with hepatitis B can get a vaccine at birth to prevent the infection	T	581 (59.8)	390 (40.2)

*Note*: *n* = 971; T = true; F = false.

### 4.4 Hepatitis B stigma endorsement

Table 3 presents participants' responses to the hepatitis B stigma endorsement index. Items on stigma most frequently endorsed were: I would not marry someone with hepatitis B (61.9% agreed), I would not allow my child to attend a school where one of the students had hepatitis B (57.2% agreed) and I would feel uncomfortable wearing a cloth once worn by someone with hepatitis B (65.8% agreed). The average stigma score was 58.44 (SD = 11.90) (range 1–5).

**TABLE 3:** Hepatitis B stigma endorsement items and scores

	Strong disagree/disagree	Neutral	Strongly agree/agree
People with hepatitis B should be isolated from others to protect the public	553 (57.0%)	12 (1.2%)	406 (41.8%)
It is not safe for people with hepatitis B to work with children	485 (49.9%)	16 (1.6%)	470 (48.4%)
People with hepatitis B should not be allowed to work in certain areas such as restaurants	393 (40.4)	18 (1.9%)	560 (57.7%)
I would feel pity for someone with hepatitis B	339 (34.9%)	31 (3.2%)	602 (61.9%)
A person with hepatitis B must have done something wrong and deserves to be sick	397 (40.9%)	18 (1.9%)	556 (57.3%)
Parents are at fault for their children getting hepatitis B	762 (78.4%)	28 (2.9%)	181 (18.6%)
People with hepatitis B should be ashamed of their illness	718 (73.9%)	30 (3.1%)	223 (23.0%)
People with hepatitis B are unclean	659 (67.9%)	23 (2.4%)	289 (29.8%)
I would not want my child to attend a school where one of the students had hepatitis B	402 (41.4%)	14 (1.4%)	555 (57.2%)
I would not want to work in an office where one of the people had hepatitis B	642 (66.1%)	14 (1.4%)	315 (32.4%)
I would not want to go to a small neighbourhood supermarket where the owner had hepatitis B	475 (48.9%)	17 (1.8%)	479 (49.4%)
I would feel uncomfortable wearing a cloth once worn by a person with hepatitis B	312 (32.2%)	20 (2.1%)	639 (65.8%)
I would feel uncomfortable sharing a meal with someone who has hepatitis B	463 (47.7%)	24 (2.5%)	484 (49.9%)
I would not want to be friends with someone with hepatitis B	564 (58.1%)	19 (2.0%)	388 (39.9%)
I would not employ someone with hepatitis B to work for me	697 (71.8%)	15 (1.5%)	259 (26.6%)
I would feel uncomfortable having a conversation with someone with hepatitis B	695 (71.6%)	10 (1.0%)	266 (27.4%)
I would not kiss someone with hepatitis B	190 (19.6%)	26 (2.7%)	755 (77.7%)
I would not date someone with hepatitis B	384 (39.5%)	16 (1.6%)	571 (58.8%)
I would not marry someone with hepatitis B	317 (32.6%)	53 (5.5%)	601 (61.9%)
I would avoid staying in the same room with someone with hepatitis B	323 (33.0%)	43 (4.4%)	605 (62.3%)

*Note*: *n* = 971.

### 4.5 Knowing someone with hepatitis B

Overall, 710 (73.1%) of the participants indicated that they knew someone with hepatitis B. The individuals known to have hepatitis B by the participants included friends (37.46%), siblings (26.90%), parents (26.20%) and extended family members (9.44%).

### 4.6 Demographic correlates of hepatitis B testing

Table 4 presents the associations between participants' demographic characteristics and hepatitis B testing using the Pearson chi‐squared test. Among the demographic characteristics only marital status (χ^2^ [3, *N* = 971] = 11.01, *p* = 0.006) and educational attainment (*χ* ^2^ [4, *N* = 971] = 18.42, *p* = 0.001) had a statistically significant association with hepatitis B testing. Specifically, 100% of the participants co‐habiting indicated that they have tested, followed by those who said they are single (54.6%), divorced (53.8%) and married (50.8%). Also, those with no formal education (65.7%) indicated that they have tested, followed by those with junior high education (58.3%), senior high education (56.6%), primary (45.5%) and post‐secondary education (44.2%).

**TABLE 4:** Association between demographic characteristics and hepatitis B testing

Variables	Hepatitis B testing				
	No, *N* = 451 (46.4%), *n* (%)	Yes, *N* = 520 (53.6), *n* (%)	Total, *N* = 971, *n* (%)	*χ* ^2^	*p* value
Gender					
Male	211 (46.2)	246 (53.8)	457 (47.1)	0.03	0.461
Female	240 (46.7)	274 (53.3)	514 (52.9)		
Age					
<25	134 (42.1)	184 (57.9)	318 (32.7)	3.62	0.082
26–35	197 (49.0)	205 (51.0)	402 (41.4)		
>35	120 (47.8)	131 (52.2)	251 (25.8)		
Marital status					
Single	252 (45.4)	303 (54.6)	555 (57.2)	11.01^**^	0.006
Married	193 (49.2)	199 (50.8)	392 (40.4)		
Divorced	6 (46.2)	7 (53.8)	13 (1.3)		
Co‐habiting	0 (0)	11 (100.0)	11 (1.1)		
Level of education					
No formal education	24 (34.3)	46 (65.7)	70 (7.2)	18.42^***^	0.001
Primary school	73 (54.5)	61 (45.5)	134 (13.8)		
Junior high school	105 (41.7)	147 (58.3)	252 (26.0)		
Senior high school	134 (43.4)	175 (56.6)	309 (31.8)		
Post‐secondary education	115 (55.8)	91 (44.2)	206 (21.2)		
Religion					
Christianity	341 (47.7)	374 (52.3)	715 (73.6)	2.89	0.118
Islam	103 (42.2)	141 (57.8)	244 (25.1)		
Traditional worship	7 (58.3)	5 (41.7)	12 (1.2)		
Occupation					
Farming	76 (42.7)	102 (57.3)	178 (18.3)	6.38	0.086
Trading	117 (42.5)	158 (57.5)	275 (28.3)		
Civil servant	125 (47.9)	136 (52.1)	261 (26.9)		
Unemployed	101 (50.5)	99 (49.5)	200 (20.6)		
Student	32 (56.1)	25 (43.9)	57 (5.9)		
Place of residence					
Greater Accra	240 (47.7)	263 (52.3)	503 (51.8)	0.67	0.225
Northern region	211 (45.1)	257 (54.9)	468 (48.2)		

*Bold indicates significant values.Note*: *n* = 971.

^**^ *p* < 0.01.

^***^ *p* < 0.001.

### 4.7 Psychosocial correlates of hepatitis B testing

In addition to the univariate analyses (Table 4), multivariate analyses, specifically binary logistic regression analyses were performed to establish factors that have unique associations with hepatitis B testing (Table 5). The findings in Model 1 showed that participants with no formal education (OR = 2.45, 95% CI: 1.39–4.31), junior high education (OR = 1.78, 95% CI: 1.23, 2.59), and senior high education (OR = 1.64, 95% CI: 1.15–2.34) were significantly more likely to have tested for hepatitis B compared to their counterparts with post‐secondary level education. The findings were similar for primary education but are not reported to ensure parsimony. Model 2 shows that having higher knowledge about hepatitis B was positively associated with hepatitis B testing (OR = 1.22, 95% CI: 1.14–1.30) and higher stigma endorsement was negatively associated with hepatitis B testing (OR = 0.97, 95% CI: 0.96–0.99). Also, participants who knew someone with hepatitis B were about 7.2 times more likely to have tested compared to their counterparts who did not know someone with hepatitis B (OR = 7.15, 95% CI: 5.04–10.14). The total variance explained was 21.0% using Cox and Snell *R* ^2^ and 28.0% using Nagelkerke *R* ^2^.

**TABLE 5:** Psychosocial correlates of hepatitis B testing

Variable	*b*	Model 1 Odds ratio	95% CI	*b*	Model 2 Odds ratio	95% CI
*Predisposing factors*						
Educational attainment^a^						
No formal education	0.90^**^	2.45	1.39, 4.31	1.02^**^	2.76	1.45, 5.27
Primary school	0.04	1.04	0.67, 1.61	0.29	1.33	0.81, 2.19
Junior high school	0.58^**^	1.78	1.23, 2.59	0.63^**^	1.87	1.24, 2.84
Senior high school	0.49^**^	1.64	1.15, 2.34	0.52^**^	1.68	1.13, 2.51
Marital status^b^						
Never married	0.16	1.17	0.91, 1.52		1.12	0.83, 1.49
Enabling and impeding factors						
Hepatitis B Knowledge				0.20^***^	1.22	1.14, 1.30
Hepatitis B Stigma					0.97	0.96, 0.99
*Need factor*						
Knows someone with HBV^c^						
Yes				1.97^***^	7.15	5.04, 10.14
Constant	−‐0.33	0.72		−2.22	0.12	
Model fitting information						
‐2Log likelihood	1320.14				1112.8	
Hosmer–‐Lemeshow χ^2^ (sig.)	2.28 (0.94)				9.72 (0.29)	
Nagelkerke *R* ^2^	0.03				0.29	

*Note*: Model 1 = Significant demographic variables following bivariate analysis.

Model 2 = All variables in model 1 plus the predictors of interest.

*n* = 971, CI, confidence interval.

^a^ Post‐secondary education is the reference category for education variable.

^b^ Ever married is the reference category for marital status variable.

^c^ No is the reference category for knowing someone with hepatitis B.

^**^ *p* < 0.01.

^***^ *p* < 0.001.

## 5 DISCUSSION

This study investigated knowledge, stigma endorsement and knowing someone with hepatitis B as correlates of hepatitis B testing among people in the Greater Accra and Northern regions of Ghana. The findings indicate that having more knowledge about hepatitis B and knowing someone with hepatitis B (i.e. parent, sibling and/or friend) were positively associated with hepatitis B testing. Additionally, more stigma endorsement was negatively related to hepatitis B testing.

In this study, knowing someone with hepatitis B was the strongest determinant of testing. This finding confirms a previous report in Zambia where close contacts of PWHB opted for testing after learning about a family member having hepatitis B (Franklin et al., 2018). In addition, a study that examined barriers to, and factors predicting, hepatitis B screening among Asian individuals in Michigan, the USA, identified knowing someone with hepatitis B as the only predictor for screening (Cheng et al., 2017). A possible reason for the higher testing behaviour of participants without a formal education compared to their counterparts is that the majority of participants with no formal education in this study knew someone who had hepatitis B. Knowing someone with hepatitis B improves hepatitis B knowledge (Jin et al. 2022) and clarifies misconceptions and tends to minimise stigmatising reactions (Cheng et al., 2017). In this present study, PWHB known by our participants were mostly immediate family members (i.e. parents, siblings) and friends. As such, it is not surprising that knowing someone with hepatitis B is linked to increased likelihood of testing. In the context of HIV, Earnshaw et al. (2012) have claimed that peoples' desire to get tested for HIV is a function of their risk perception: people who feel they are at risk are more likely to engage in testing (Earnshaw et al., 2012). We believe that participants who were close to someone with hepatitis B perceived greater risk and thus more likely to test. The finding also suggests that openness about having hepatitis B can both reduce stigma and encourage more testing, which subsequently leads to new diagnoses (WHO, 2017b).

In our study, we also found a positive association between hepatitis B knowledge and hepatitis B testing. The finding corroborates previous studies conducted in Canada (Li et al., 2012) and the United States (Calderon et al., 2014) where more knowledge about hepatitis B was related to higher testing uptake. For example, in a clustered‐randomised trial that involved Korean church members in Los Angeles, participants with improved knowledge on liver cancer and hepatitis B testing were nearly five times more likely to get tested for hepatitis B (Bastani et al., 2015). In our study, most participants demonstrated fair knowledge about hepatitis B. More than three‐quarters of the participants inaccurately indicated that hepatitis B can be transmitted through sweat. There is no evidence that hepatitis B transmission can be transmitted through sweat (Schillie et al., 2018). This misconception has previously been observed in Ghana (Adjei et al., 2018, 2019) and we believe that the confusion might be the result of earlier hepatitis B awareness campaigns in Ghana that focused on bodily fluids in a more general sense as a means of hepatitis B transmission.

We further found that higher hepatitis B stigma endorsement was associated with less testing. The negative association between stigma endorsement and testing found is consistent with findings from the US, Canada, the Netherlands and Zambia (Franklin et al., 2018; Hamdiui et al., 2018; Hara et al., 2018; Li et al., 2012; Sriphanlop et al., 2014). This is also as expected, particularly in light of the fact that Ghanaians tend to have high stigma consciousness (Kwabea Owusu‐Daaku & Buanya‐Mensah, 2010). Previous research has shown that two main factors contribute to hepatitis B stigma in Ghana: 1) the pervasive misconception that hepatitis B is dangerous and deadly; and 2) the perception that hepatitis B is highly infectious and transmissible through casual contacts (Adjei et al., 2019). Additionally, a recent report showed that, in Ghana, hepatitis B stigma is prevalent in schools, workplaces and healthcare settings (Adjei et al., 2019). As such, it is plausible that a number of participants in this study had witnessed or exhibited a stigmatisation of PWHB in the past and feared being the recipient of such stigmatisation should they test positive for hepatitis B.

Regarding testing, approximately 54% of our participants self‐reported having tested for hepatitis B. This sub‐optimal uptake of hepatitis B testing may be the result of hepatitis B activities by Non‐Governmental Organisations (NGOs). In Ghana, NGOs are often engaged in hepatitis B testing in churches, mosques, lorry stations and market places in urban areas (Adjei et al., 2017).

### 5.1 Theoretical and practical implications

This study has some important theoretical and practical implications. In terms of theory, this study adds to the limited number of quantitative studies on the correlates of hepatitis B testing in Africa. In the context of our theoretical model, namely the behavioural model of health service utilisation, it affirms knowledge as an enabling factor, stigma as an impeding factor and knowing someone with hepatitis B as a need factor that influences hepatitis B testing in the Ghanaian context. In terms of practice, based on our finding that stigma endorsement is negatively related to testing, we recommend stigma reduction interventions in Ghana. This is particularly important also in light of our finding that knowing someone with hepatitis B is positively related to testing as stigma may hinder disclosure. There is therefore also a need to create safe and non‐judgmental contexts for PWHB to disclose if they want to. Importantly, disclosure should be preceded by hepatitis B education particularly for family members so that they can better understand the modes of hepatitis B transmission as well as prevention methods (i.e. vaccination) to avoid stigmatisation following disclosure of hepatitis status.

### 5.2 Limitation of the study

Our study has some limitations. First, the use of the cross‐sectional study design limits the possibility of establishing causal relationships. Second, we acknowledge the possibility of gambling in our participants' response to the knowledge items. The extent of gambling was however reduced by including the ‘do not know’ option. Third, given that the total variance explained by our variables of interest on testing was relatively low, that is 21.0% using Cox and Snell *R* ^2^ and 28.0% using Nagelkerke *R* ^2^, we recommend that future research further investigate other possible correlates of testing such access to testing (e.g. distance to testing site), social norms for testing and self‐efficacy. Accordingly, we recognise that the operationalisation of the behavioural model of health service utilisation by our study is limited in scope given that there is other possible predisposing, enabling, impeding and need factors that were not examined. Finally, the convenience sampling technique imposes some limitations on the study's findings as it does not ensure randomisation, thereby limiting the possibility of generalising the results to the entire regions of Ghana.

## 6 CONCLUSIONS

This study demonstrates that knowing someone with hepatitis B increases the likelihood of testing and this points to the need to create safe and non‐judgmental contexts for PWHB to disclose if they want to. Also, given that higher knowledge about hepatitis B increases testing and hepatitis B stigma endorsement impedes testing, interventions that aim to increase knowledge and reduce hepatitis B stigma should be included in efforts to encourage testing in Ghana.

## AUTHOR CONTRIBUTIONS

CAA, SS, RR and FN was involved in the conceptualization of the study. Data analyses and interpretation and the writing of the manuscript were done by CAA, FB, SS, RR and FN.

## ETHICAL APPROVAL

Ethical clearance was obtained from Institutional Review Board of Korle‐Bu Teaching Hospital (Approval number KBTH‐IRB 00092/2016). Permission was sought from the management of the data collection sites, and informed consent (written) was obtained from the participants.

## CONFLICT OF INTEREST

The authors declare no conflict of interest in this study.

## Data Availability

All data are with the authors and available for sharing on request.
